# Accelerated Orthodontics: Stepping Into the Future Orthodontics

**DOI:** 10.7759/cureus.46824

**Published:** 2023-10-11

**Authors:** Dolly Gabada, Amit Reche, Kaushiki P Saoji, Radha Deshmukh, Netal Rathi, Achal Mantri

**Affiliations:** 1 Public Health Dentistry, Sharad Pawar Dental College, Datta Meghe Institute of Higher Education and Research, Wardha, IND; 2 Musculoskeletal Physiotherapy, Ravi Nair Physiotherapy College, Datta Meghe Institute of Higher Education and Research, Wardha, IND

**Keywords:** periodontics, orthodontics, laser, corticotomy, piezocision, wilckodontics

## Abstract

Orthodontic treatment signifies a transformative journey for individuals seeking not only enhanced oral health but also a boost in aesthetics and self-confidence. Nonetheless, the protracted timeline associated with conventional orthodontic care has been a persistent concern for both patients and practitioners. In this comprehensive review, we embark on an exploration of innovative strategies aimed at expediting orthodontic tooth movement (OTM). By doing so, we aspire to curtail treatment duration and mitigate potential risks, ultimately culminating in an elevated and more fulfilling patient experience.

Traditionally, orthodontists heavily leaned on surgical techniques to hasten tooth movement. However, the recent landscape of orthodontics has been profoundly shaped by technological advancements and groundbreaking research findings, ushering in an era characterized by the embrace of minimally invasive approaches. These progressive methodologies encompass procedures like Piezocision, Discision, and Microosteoperforation (Alveocentesis). Beyond the obvious benefits of reduced patient discomfort, these techniques significantly truncate treatment periods, a development that addresses a primary concern of many patients. Moreover, this review delves into non-invasive alternatives, including cyclic vibrations, photobiomodulation, direct light electric current, and static or pulsed magnetic fields, as well as systemic and local administration of biological substances and hormones, all of which hold substantial promise in optimizing OTM.

Furthermore, our exploration extends to a diverse spectrum of medications that have demonstrated their efficacy in expediting OTM. These encompass NSAIDs, acetaminophen, corticosteroids, bisphosphonates, herbal medicine biomaterials, and synthetic biomaterials like graphene dioxide. Every technique and medication is subjected to meticulous evaluation, taking into account its indications, contraindications, advantages, disadvantages, clinical implications, and limitations. Through this review, we endeavor to equip orthodontic professionals with a profound understanding of these innovative strategies. By doing so, we enable them to make informed decisions, tailored meticulously to meet the unique needs of each patient. In an ever-evolving field like orthodontics, staying abreast of these advancements becomes paramount, ultimately contributing to heightened treatment efficacy and enhanced patient satisfaction. The adoption of these innovative strategies not only holds the potential for improved clinical outcomes but also champions a patient-centric approach that could fundamentally reshape the landscape of orthodontic care.

## Introduction and background

The lengthiest dental procedure ever conducted, in terms of time, may be orthodontic treatment. Numerous patients-particularly adults-are prompted by lengthy orthodontic treatment to forego receiving it altogether or to look for less effective, less permanent alternatives. Prolonged orthodontic treatment may also be associated with longer treatment time and various adverse effects such as increased decalcification, root resorption, dental cavities, gingival irritation, temporomandibular disorders, etc. It has long been a goal of orthodontics to accelerate tooth movement since it may have several advantages, including a shorter treatment time, better post-treatment stability, and fewer negative effects from prolonged therapy. When an orthodontic force is applied to a tooth, a variety of mechanical, chemical, and biological processes occur in the paradental tissues, producing architectural changes that promote tooth movement. Enhancing the body's reaction to orthodontic stresses can shorten treatment time and hasten tooth movement. Accelerating tooth movement has been attempted since the 1890s, it almost exactly corresponds to Angle's revolutionary work in modern orthodontics. Today, there are several ways to perform rapid orthodontics, including invasive, minimally invasive, and non-invasive methods. It has been successfully demonstrated that these techniques can cut treatment times by up to 70% [1]. In the periodontal ligament (PDL), orthodontic force causes a biological reaction that results in bone resorption on the pressure side and bone deposition on the tension side. This is caused by the receptor activator of nuclear factor kappa-B-receptor activator of nuclear factor kappa-B ligand (RANK-RANKL) pathway's stimulation of osteoclasts as well as the presence of several inflammatory mediators, including TNF-alpha, IL-1, and IL-8 [2,3]. To hasten tooth movement, surgical techniques have been employed for a long time. These techniques rely on the idea that surgically irritating the bone would set off an inflammatory cascade that would promote osteoclastogenesis and hasten tooth movement (regional acceleratory phenomenon (RAP) or periodontally accelerated osteogenic orthodontics (PAOO)) [4]. These, however, were invasive, and patients did not take them well. Hence newer surgical methods were introduced.

Historical background

Over the past century, various surgical procedures to alter alveolar housing and subsequently tooth movement have been documented. The first person to describe corticotomy-facilitated orthodontics was Heinrich Kole in 1959 [5]. Kole believed that the continuous, thick layer of the thicker cortical bone provided the best defense against the rapid movement of the teeth, he claimed that “blocks of bone” were responsible for the selective corticotomy-induced acceleration of tooth movement. In contrast to Kole's notion, Wilcko observed in 2001 that a temporary localized demineralization-remineralization mechanism in the bony alveolar housing was responsible for accelerated tooth movement. He combined advanced corticotomy-facilitated orthodontic surgery with alveolar augmentation using particulate bone transplant [6]. In 2004, Cruz et al. conducted the first study on humans to examine how low-intensity laser therapy affected the movement of orthodontic teeth. They demonstrated that, over a 60-day period, the irradiated dogs retreated 34% faster than the control dogs [7]. Park et al. devised a different surgical procedure called “corticision” in 2006 that was thought to be a micro-invasive method to accelerate tooth movement [8]. In order to facilitate quick tooth movement, Vercelotti and Podesta first used piezosurgery in conjunction with standard flap elevations in 2007 [9]. Using a piezoelectric knife, Dibart et al. in 2009 described piezocision, which left the palate or lingual cortex unaffected [10]. Alveocentesis, a new micro-invasive procedure, was unveiled in 2011 that accelerates alveolar bone rebuilding by cytokine activity. The PROPEL system, a novel approach to accelerate alveolar bone remodeling, has been created and patented. To move the teeth into the clinically desirable position more consistently and quickly, the PROPEL system's microosteoperforations (MOPs) trigger cytokines in the bone [11].

## Review

Search methodology

In May 2023, we conducted a review using the terms rapid orthodontics, Wilckodontics, Piezocision, Corticotomy, Laser, Orthodontics, and periodontics in PubMed and Google Scholar. Additionally, we looked through the bibliographies of pertinent research to find important references. In July 2023, the search was updated. The reviewer initially checked the titles and abstracts of the retrieved papers against the inclusion and exclusion criteria before checking the full texts. Both published and unpublished studies in the English language were taken into consideration for inclusion. Due to a lack of resources and the reviewer's inability to access full-text articles, we excluded research that was published in other languages. Some of the current commonly used techniques for accelerating orthodontic tooth movements (OTMs) are listed (Table 1).

**Table 1:** Classification of some current common techniques for accelerating tooth movement

Category	Subcategory	Technique
Invasive Techniques		Interseptal alveolar surgery (Distraction Osteogenesis)
		Conventional Corticotomy
		PAOO (Wilckodontics)
Minimally Invasive Techniques		Piezocision & Discision
		Microosteoperforation (Alveocentesis)
Non-invasive Techniques	Device-assisted treatment	Cyclic Vibrations
		Low-Level Laser Therapy (Photobiomodulation)
		Direct light electric current
		Static or pulsed magnetic field
	Biologic Approach	Systemic and local administration of biological substances and hormones such as Parathyroid hormone, prostaglandins, thyroxine, and calcitonin, relaxin, 1,25 dihydroxycalciferol (vit D3 or calciferol)
		Neurotransmitters
	Medications	NSAIDS
		Acetaminophen (paracetamol)
		Corticosteroids
		Bisphosphonates
		Herbal medicine biomaterials (Asperosaponin VI)
	Synthetic Biomaterials	Graphene oxide

Invasive techniques

Interseptal Alveolar Surgery (Distraction Osteogenesis)

Interseptal alveolar surgery, also referred to as subperiosteal osteotomy, is a highly controlled procedure involving the gradual displacement of fractures induced through medical means. This technique leads to simultaneous growth in both soft tissue and bone volume by mechanically straining the osteotomy site. This, in turn, has the potential to result in the distraction of the PDL or the dentoalveolar bone [12].

Procedure: Typically, this procedure is carried out during premolar extraction, with a focus on the interseptal bone located distal to the canine. Surgical undermining is performed to prepare the area for the procedure. A custom-made stainless steel intraoral, tooth-borne device serves as the distraction tool. It is important to note that interseptal alveolar surgery replaces compact bone with woven bone, which is known to be less resistant to tooth movement. The initial stages of tooth movement, particularly within the first week following the procedure, demonstrate rapid movements [13]. For a more detailed understanding of the indications and contraindications associated with this procedure (Table 2) [12,13].

**Table 2:** Various indications and contraindications of distraction osteogenesis

Indications	Contraindications
Unilateral and bilateral craniofacial microsomia	Poor nutrition
Developmental micrognathia	Lack of soft tissue
Mandibular condyle regeneration	Irradiated bone
Correction of mild skeletal class II	Osteoporotic bone
Rapid Canine retraction	Systemic diseases affecting bone metabolism
TMJ Ankylosis

Corticotomy

During a corticotomy surgical procedure, the primary focus lies on the mechanical alteration, cutting, or perforation of the cortical bone. Unlike osteotomies, which involve the complete thickness of the bone, corticotomy surgery is specifically directed at the cortical bone, leaving the medullary bone untouched [5]. A comprehensive overview of the indications, and contraindications, as well as the advantages and disadvantages associated with this procedure can be found in Table 3 [5,14-16].

**Table 3:** Various indications, contraindications, advantages, and disadvantages of corticotomy

Category	Description
Indications	Used to accelerate canine retraction following extraction of the premolars
	improving post-orthodontic stability
	To facilitate slow orthodontic expansion.
Contraindications	Patients with active periodontal infection
	Patients with gingival recession
	patients having Bimax protrusion with gummy smile
Advantages	Slight changes in periodontium occur
	Bone augmentation can be done
	periodontal defect can be avoided
	Faster tooth movement occurs in less time
	Root resorption is less
Disadvantages	Invasive procedure
	Expensive
	may result in postoperative pain and swelling

Procedure: To perform this procedure effectively, the surgeon begins by raising full-thickness buccal and/or lingual mucoperiosteal flaps. Precise corticotomy cuts are meticulously placed while ensuring continuous irrigation, which is achieved using specialized tools such as a Piezosurgical device or a micromotor. In cases where it is deemed necessary, graft material may be introduced into the corticotomy sites to enhance bone thickness and support structural integrity [14]. What makes corticotomies particularly intriguing is their impact on orthodontic movements, which can be rendered more efficient due to the trabecular bone structure's response to the biological stimulation induced by corticotomies [15]. When performed with meticulous patient selection, corticotomies emerge as a potent and secure method to significantly enhance the efficacy and efficiency of orthodontic treatments [16]. The precise customization of this procedure presents a promising avenue for optimizing orthodontic care for eligible patients.

PAOO, Wilckodontics

A groundbreaking technique, initially known as “accelerated osteogenic orthodontics” and later referred to as “periodontally accelerated osteogenic orthodontics (PAOO),” was introduced by Dr. Thomas J. Wilcko and Dr. William M. Wilcko in 2001 through a fusion of corticotomy and alveolar bone grafting [6]. PAOO is an innovative approach in orthodontics that aims to expedite tooth movement and enhance the outcomes of orthodontic treatment. This technique capitalizes on the RAP, an observed healing event named by Frost in the 1980s, which facilitates rapid tooth movement [16].

The mechanism behind RAP involves increased metabolic activity, heightened blood flow, and enhanced cellular activity in the tissues surrounding an injury or surgical site. This heightened activity triggers the activation of osteoclasts and osteoblasts, the cells responsible for bone remodeling, leading to accelerated bone turnover and facilitating tooth movement. Notably, the healing process following corticotomies paves the way for accelerated recovery, occurring 2-10 times faster than typical physiological repair [6]. PAOO offers distinct advantages, including increased alveolar bone volume and the potential to address previous bony dehiscence and fenestrations through various bone grafts [6].

The duration of the RAP phenomenon varies from several weeks to a few months following the procedure, depending on factors such as the type and extent of treatment, individual patient characteristics, and the specific tissues involved. Moreover, the timing of orthodontic force application is critical after PAOO surgery, with a recommended delay of no longer than two weeks [6]. This comprehensive approach is accompanied by a range of indications, contraindications, and a thorough assessment of its advantages and disadvantages, all detailed in Table 4 [6,16]. PAOO represents a remarkable stride in orthodontics, capitalizing on the body's natural healing processes to expedite orthodontic treatment while rectifying bone-related issues, ultimately providing patients with a promising and efficient treatment option.

**Table 4:** Various indications, contraindication, advantages, and disadvantages of PAOO

Category	Description
Indications	Class I malocclusion with moderate to severe crowding
	Class II malocclusion which requires expansion or extraction
	Class III mild cases.
Contraindications	Prolonged history of corticosteroid use
	Patients presenting with active periodontal disease
	Patients using drugs like NSAIDS or bisphosphonates that affect how bone metabolism
Advantages	Post orthodontic stability is increased
	Numerous authors have found it effective to speed up tooth movement
	Bone augmentation can be done, which prevent periodontal defect occurring due to thin alveolar bone
Disadvantages	Increased rate of morbidity
	Postoperative complications such as pain, swelling, infection, and vascular necrosis might occur
	Patient compliance is less
	Vital structures might get damaged

Minimally invasive technique

Piezocision

Piezocision, introduced as a less invasive alternative to full-thickness flap reflection for corticotomy, represents a significant advancement in orthodontic procedures [10]. Proposed by Dibart et al. in 2009, this flapless corticotomy technique harnesses Piezosurgery to create buccal cortex osseous cuts through a series of micro-incisions that solely affect the buccal gingiva, without compromising the palatal or lingual cortex [10]. This approach effectively addresses the drawbacks of time-consuming and traumatic surgical methods while enabling swift tooth movement. Furthermore, it maintains the therapeutic benefits of concurrent soft tissue or grafting treatments through a tunnel technique [10,17,18]. Combining Piezocision with Invisalign has not only enhanced aesthetics but also improved efficiency [19].

In the realm of orthodontics, Piezocision offers remarkable advantages, with a range of indications, contraindications, and a meticulous assessment of its pros and cons outlined in Table 5 [10,17-19]. One of the most significant implications of Piezocision is its ability to facilitate rapid OTM while mitigating the drawbacks associated with traditional surgical approaches, making it a preferred choice for various procedures, including the distalization of molars and root intrusion with lingual torque [20]. Its low morbidity enables the reactivation of the RAP when needed, further enhancing its appeal to both patients and orthodontic professionals. In summary, Piezocision presents a compelling case for wider acceptance within the dental and patient communities due to its effectiveness and patient-centric benefits [20].

**Table 5:** Various indications, contraindications, advantages, and disadvantages of the Piezocision

Category	Description
Indications	Anterior crowding
	Anterior open bite
	Deep bite correction
	Class II (Endon) cases
Contraindications	Patients/operators with pacemakers or any active implants
	Mixed dentition
	Medically compromised patients
Advantages	Minimally invasive
	Postoperative discomfort is less
	Permits simultaneous soft-tissue and/or hard-tissue augmentation
	Patient compliance is better
Disadvantages	Increased risk of root damage

Microosteoperforations

Propel Orthodontics introduced a revolutionary device known as “PROPEL,” designed for accelerated orthodontic movement with minimal tissue invasion in the surrounding bone tissues. MOPs performed by this device stimulate inflammatory markers, enhancing osteoclastic activity and expediting tooth movement. This sterilized, ready-to-use disposable device features an adjustable depth dial, offering settings of 0 mm, 3 mm, 5 mm, or 7 mm tip depth, tailored to the specific treatment area [21].

Procedure: The process entails creating tiny perforations, approximately 0.25 mm in size, through the cortical bone in the premolar and molar area using a round bur and handpiece after gently reflecting a soft tissue flap [21]. Typically, several such perforations, usually four to six, are made on both aspects of the tooth to facilitate accelerated tooth movement [21]. The advantages of this procedure include its minimally invasive nature, repeatability, and compatibility with standard orthodontic appliances, eliminating the need for specialized instruments [21].

While MOPs can enhance osteoclast activity and expedite tooth movement, it is important to understand that they may not completely mitigate the negative impacts on the biomechanical plan. In cases where there isn't enough space, teeth may struggle to engage with the main archwire, similar to traditional orthodontic mechanics. MOPs are most valuable as a supplementary treatment for protracted or retracted teeth or groups of teeth. They result in a decrease in bone density between tooth roots while maintaining consistency around anchor teeth. Consequently, this treatment proves particularly beneficial when moving a tooth into an edentulous area with dense alveolar bone and an average ridge. It is advisable to repeat the MOPs procedure approximately two months after the initial application, as the heightened cytokine activity tends to decline over time. However, it is crucial to exercise caution when applying MOPs near temporary anchorage devices (TADs) to avoid potential bone density loss and reduced TAD stability [22].

Non-invasive techniques

Device-Assisted Treatment

Cyclic vibrations: Cyclic vibrations, as a technique, involve the application of gentle alternating pressures to the teeth through mechanical radiations. This approach has demonstrated its effectiveness by eliciting in vitro cellular responses to mechanical stress within a remarkably short timeframe of 30 minutes [23]. The vibration-imposed device utilized for this purpose comprises essential components such as a vibration controller, charge amplifier, vibrator, force sensor, and accelerometer. Signals from both the accelerometer and force sensor are transmitted to the vibration controller, which subsequently excites the vibrator through the amplified sound. This ensures that the acceleration is consistently maintained at 1.0 meters per square second (m/s^2) by delivering controlled vibrations via the power amplifier, guided by the output signal from the accelerometer. The attachment of the vibrator's tip to the tooth is facilitated with the aid of adhesive. During the vibration experiments, which typically last for five minutes, resonance curves depicting frequency-force relationships are displayed on the vibration controller's monitor. Clinical studies involving the general population have seen the utilization of oral vibrating tools such as AccleDent TM, AcceleDent, and electric toothbrushes. These studies have consistently shown the effectiveness of these tools in accelerating tooth movement [1,24-27]. Additionally, two cross-sectional animal experiments have been conducted to explore the impact of cyclic vibration in greater detail [1,24].

AcceleDent: AcceleDent is a user-friendly device equipped with a mouthpiece designed to seamlessly fit over your existing braces. Its daily usage involves activating it for 20 minutes, generating gentle vibrations [28]. This portable device features a charging connector that operates akin to other electrical devices, ensuring convenience and ease of use. This treatment approach boasts several advantages, including a diminished risk of root resorption, reduced instances of mini-implant failure, and notably, improved patient compliance. However, it is essential to acknowledge that a notable drawback is the requirement for a specialized and relatively costly instrument, which contributes to an increase in the overall treatment cost [23-25].

Low-level laser therapy (LLLT) (photobiomodulation): Photobiomodulation therapy, commonly referred to as LLLT, has emerged as one of the most effective methods for expediting tooth movement. Laser light plays a pivotal role in influencing bone remodeling and accelerating tooth movement by promoting the growth of key players in bone physiology, including osteoclasts, osteoblasts, and fibroblasts [29]. The underlying mechanisms facilitating this accelerated tooth movement encompass the production of ATP, activation of cytochrome C, and the expression of critical factors such as RANK/RANKL, macrophage colony-stimulating factor, and its receptor [30,31].

Extensive research conducted by various investigators has consistently demonstrated the potential of LLLT to expedite tooth movement [32-36]. Notably, Limpanichkul et al. identified a specific LLLT setting (25 J/cm^2^) that may have fallen below the threshold for eliciting either a stimulatory or inhibitory effect on the rate of OTM in their study, resulting in inconclusive outcomes [33]. Discrepancies in laser frequency, intensity, and application methods appear to underlie the variations observed across different studies. Among the six studies conducted on LLLT, five comprised randomized controlled trials involving human subjects [33-36], while one encompassed a cross-sectional animal study [32]. A recent clinical investigation found that using an 800 nm continuous wave laser operating at an output of 0.25 mW, with an exposure time of 10 seconds, increased tooth movement by 1.3-fold, highlighting the promising potential of LLLT in orthodontic treatments [37].

Biological Approach

This encompasses the systemic or local administration of biological substances, hormones, neurotransmitters, and medications field extensively explored in OTM acceleration research. These attributes have undergone thorough scrutiny in studies, particularly in animal models, yielding diverse outcomes. Orthodontic pressures, by causing matrix and cell distortion and inducing fluid movement within the PDL space, initiate the process of bone remodeling, crucial for facilitating tooth movement. Several studies have delved into pharmacological substances functioning as biomodulators to promote OTM. Among the most frequently employed pharmacological agents for expediting tooth movement and reducing treatment durations are parathyroid hormone (PTH), Vitamin D, Prostaglandins, and Relaxin [1].

Parathyroid hormone: PTH, a primary regulator of bone remodeling and calcium homeostasis in the human body, plays a pivotal role in calcium reabsorption from the small intestine, thereby elevating blood calcium levels [38]. This mechanism leads to calcium ion absorption from bone, triggering bone resorption. Studies conducted on rodents by Soma and colleagues have revealed that the continuous administration of PTH can effectively expedite OTM [39,40]. Furthermore, various case-control studies in animals have been conducted to assess the role of parathyroid hormone in accelerating OTM [39,41].

Prostaglandins: Among the extensively researched substances, prostaglandin E (PGE2) stands out prominently. Numerous studies have demonstrated its impact on OTM. PGE2 has been found to increase bone resorption directly by affecting osteoclasts, leading to enhanced OTM when administered exogenously over a prolonged period. However, it is important to note that earlier studies revealed a potential downside to PGE2. Local injections of PGE2, especially in varying amounts and concentrations, when used in isolation, were associated with root resorption. Interestingly, researchers later discovered that when combined with calcium, PGE2 treatment not only stabilized root resorption but also accelerated the overall process of OTM [42,43].

Thyroxine and calcitonin: Thyroxine and calcitonin, two vital thyroid hormones, play a pivotal role in calcium reabsorption. The administration of thyroxine has been observed to increase bone remodeling activity while concurrently reducing bone density. Thyroxine also triggers the production of Interleukin 1 (IL-1B), a cytokine with a significant role in osteoclastic response and bone production. When used locally, thyroxine has the remarkable ability to expedite OTM by stimulating osteoclasts, contributing to the overall efficiency of orthodontic treatment [44].

Relaxin: Relaxin, a naturally occurring hormone primarily recognized for its role in widening pubic ligaments after childbirth, boasts additional functionalities. It influences collagen turnover, angiogenesis, and anti-fibrosis processes. In a notable rat study, local administration of human relaxin was found to accelerate OTM. However, this acceleration came at a cost, as it altered the degree of PDL organization and reduced mechanical strength. These effects culminated in increased tooth mobility, an important consideration when exploring relaxin's potential in orthodontic treatment [45].

1,25 dihydroxycholecalciferol (vitamin D3 or calcitriol): In an in vivo study on dogs, locally administered calcitriol was found to significantly accelerate OTM. The dose of 25 pg calcitriol resulted in an almost 51% faster rate compared to the control side, while doses of 15 pg and 40 pg each led to approximately 10% accelerated OTM. Notably, periapical radiographs showed no detrimental effects of calcitriol on surrounding tissues. In comparisons between local injections of PGE2 and vitamin D in rat groups, no significant difference in acceleration was observed. However, the vitamin D injection group had more osteoblasts on the pressure side, suggesting that vitamin D might enhance bone turnover more effectively [28,46].

Neurotransmitters: Dental and periodontal tissues receive innervation from neurons of the trigeminal ganglion, which contain neuropeptides like substance P, calcitonin gene-related peptide (CGRP), and vasoactive intestinal polypeptide (VIP). These neuropeptides remain dormant in a neutral environment but become active and are released during orthodontic treatment when mechanical forces are applied. This triggers local inflammation and the release of neuropeptides, which have an immediate impact on bone remodeling and can increase vascular permeability [44,33].

Medications

NSAID: NSAIDs are used in orthodontic treatment to alleviate pain and discomfort following the application of mechanical stress to the teeth. Research consistently shows that NSAIDs reduce the incidence of OTM, with outcomes varying based on the dose and frequency of administration, despite being based on short-term administrations. NSAIDs limit tooth movement by inhibiting the inflammatory response caused by PGs, with cyclooxygenase (COX) activity playing a key role in regulating the process and altering vascular and extravascular matrix remodeling [47].

Acetaminophen (Paracetamol): Despite sharing a similar chemical composition with NSAIDs, acetaminophen does not possess anti-inflammatory properties. Extensive studies have consistently shown that paracetamol has no significant adverse impact on the rate of OTM. This makes it a widely favored and safe choice for pain management during orthodontic treatment [44].

Corticosteroids: Corticosteroids have the potential to influence bone development and expedite tooth movement. However, they come with a drawback: they inhibit the formation of new bone. This can lead to a reduction in the stability of tooth movement and, consequently, a lower success rate for orthodontic therapy [47].

Bisphosphonates (BPNs): BPNs directly affect bone metabolism and calcium homeostasis. Research findings suggest that BPNs can indeed hinder the progress of OTM, resulting in delays. Nevertheless, it is worth noting that localized administration of BPNs may offer certain advantages, especially in terms of anchoring and maintaining the position of teeth during orthodontic treatment [47].

Herbal medicine biomaterials (Asperosaponin VI): Asperosaponin VI (ASA VI), the primary component in Dipsacus asper Wall extract, a popular Chinese herbal treatment, has been shown to increase bone density and the number of trabeculae, improving bone histomorphology. Rats receiving local, submucoperiosteal, and buccal injections of ASA VI exhibited significantly higher OTM compared to the control group. ASA VI stimulates bone resorption on the pressure side while encouraging bone deposition on the tension side, evident through increased bone density and trabecular spacing [48].

Synthetic biomaterials (graphene oxide): A biocompatible form of graphene oxide, reduced by gelatin to enhance solubility, was developed by Jiao et al. In vitro research demonstrated that this functionalized biomaterial exhibited good biocompatibility. Furthermore, it was shown that when used appropriately, gelatin-reduced graphene oxide (GOG) creates a localized hypoxic environment that promotes the differentiation of mesenchymal stem cells into angiogenic cells, thus enhancing bone and tissue regeneration. Rats receiving GOG submucosally displayed higher OTM, driven by increased osteoclastogenesis, osteoblastogenesis, and angiogenesis, followed by rapid osteogenesis, compared to those given PBS [49].

## Conclusions

This comprehensive review delves into a multitude of techniques designed to expedite OTM, providing orthodontic professionals with a highly valuable resource for optimizing treatment strategies. Encompassing invasive methods such as interseptal alveolar surgery and corticotomy, as well as less invasive options like Piezocision and MOPs and extending to non-invasive approaches involving device-assisted treatments and the biological administration of substances, hormones, and medications, this article presents a diverse array of tools to elevate orthodontic practice.

The review underscores the pivotal role of tailoring treatment choices to align with individual patient needs and desired outcomes, highlighting the dynamic nature of orthodontics and the imperative for orthodontic professionals to remain abreast of the latest advancements in the field. As research and clinical investigations continue to evolve, these techniques are poised to undergo further refinement and expansion, providing orthodontists with a versatile toolkit to enhance patient experiences, curtail treatment durations, and optimize overall treatment outcomes. Remaining attuned to these innovations will empower orthodontic practitioners to proffer their patients a spectrum of options, ensuring more efficient and comfortable orthodontic journeys while propelling the field of orthodontics into the future.

## References

[1] Virdi G, Prashar A, Kaur S (2021). Accelerated orthodontics: getting ahead of ourselves. Int J Health Sci.

[2] Taddei SR, Andrade I Jr, Queiroz-Junior CM (2012). Role of CCR2 in orthodontic tooth movement. Am J Orthod Dentofacial Orthop.

[3] Uematsu S, Mogi M, Deguchi T (1996). Interleukin (IL)-1 beta, IL-6, tumor necrosis factor-alpha, epidermal growth factor, and beta 2-microglobulin levels are elevated in gingival crevicular fluid during human orthodontic tooth movement. J Dent Res.

[4] Frost HM (1983). The regional acceleratory phenomenon: a review. Henry Ford Hosp Med J.

[5] Köle H (1959). Surgical operations on the alveolar ridge to correct occlusal abnormalities. Oral Surg Oral Med Oral Pathol.

[6] Wilcko WM, Wilcko MT, Bouquot JE, Ferguson DJ (2001). Rapid orthodontics with alveolar reshaping: two case reports of decrowding. Int J Periodontics Restorative Dent.

[7] Cruz DR, Kohara EK, Ribeiro MS, Wetter NU (2004). Effects of low-intensity laser therapy on the orthodontic movement velocity of human teeth: a preliminary study. Lasers Surg Med.

[8] Park YG, Kang SG, Kim SJ (2006). Accelerated tooth movement by corticision as an osseous orthodontic paradigm. Kinki Tokai Kyosei Shika Gakkai Gakujyutsu Taikai Sokai.

[9] Vercellotti T, Podesta A (2007). Orthodontic microsurgery: a new surgically guided technique for dental movement. Int J Periodont Restorative Dent.

[10] Dibart S, Keser E (2014). Minimally invasive periodontally accelerated orthodontic tooth movement procedure. Piezocision.

[11] Nicozisis J (2012). Accelerated orthodontics with Alveocentesis. Princet Orthod Clin Orthod.

[12] Mathews DP, Kokich VG (2013). Accelerating tooth movement: the case against corticotomy-induced orthodontics. Am J Orthod Dentofacial Orthop.

[13] Lv T, Kang N, Wang C, Han X, Chen Y, Bai D (2009). Biologic response of rapid tooth movement with periodontal ligament distraction. Am J Orthod Dentofacial Orthop.

[14] Adusumilli S, Yalamanchi L, Yalamanchili PS (2014). Periodontally accelerated osteogenic orthodontics: an interdisciplinary approach for faster orthodontic therapy. J Pharm Bioallied Sci.

[15] Patil S, Bhola N, Kambala R (2022). Comparative assessment of corticotomy facilitated rapid canine retraction using piezo versus bur: a randomized clinical study. Authorea.

[16] Hassan AH, Al-Saeed SH, Al-Maghlouth BA, Bahammam MA, Linjawi AI, El-Bialy TH (2015). Corticotomy-assisted orthodontic treatment: a systematic review of the biological basis and clinical effectiveness. Saudi Med J.

[17] Sebaoun JD, Surmenian J, Dibart S (2011). [Accelerated orthodontic treatment with piezocision: a mini-invasive alternative to conventional corticotomies]. Orthod Fr.

[18] Keser EI, Dibart S (2011). Piezocision-assisted Invisalign treatment. Compendium.

[19] Talla R, Kamble R, Dargahwala H, Banerjee S (2020). Acclerated orthodontics-a review. Eur J Mol Clin Med.

[20] Yi J, Xiao J, Li Y, Li X, Zhao Z (2017). Efficacy of piezocision on accelerating orthodontic tooth movement: a systematic review. Angle Orthod.

[21] Alikhani M, Raptis M, Zoldan B (2013). Effect of micro-osteoperforations on the rate of tooth movement. Am J Orthod Dentofacial Orthop.

[22] Alikhani M, Alansari S, Sangsuwon C (2015). Micro-osteoperforations: minimally invasive accelerated tooth movement. Seminars Orthodont.

[23] Kulshrestha R, Bhalerao S, Vibhute P, Patil C, Umale V, Balgangadhar Balgangadhar (2019). Accelerated Orthodontics: A Review. J Trends Oral Health Care.

[24] Nishimura M, Chiba M, Ohashi T, Sato M, Shimizu Y, Igarashi K, Mitani H (2008). Periodontal tissue activation by vibration: intermittent stimulation by resonance vibration accelerates experimental tooth movement in rats. Am J Orthod Dentofacial Orthop.

[25] Kau CH, Nguyen JT, English JD (2010). The clinical evaluation of a novel cyclical force generating device in orthodontics. Orthod Pract US.

[26] Pavlin D, Anthony R, Raj V, Gakunga P (2015). Cyclic loading (vibration) accelerates tooth movement in orthodontic patients: a double-blind, randomized controlled trial. Seminars Orthodont.

[27] Leethanakul C, Suamphan S, Jitpukdeebodintra S, Thongudomporn U, Charoemratrote C (2016). Vibratory stimulation increases interleukin-1 beta secretion during orthodontic tooth movement. Angle Orthod.

[28] Shingade M, Maurya R, Mishra H, Singh H, Agrawal K (2017). Accelerated orthodontics: a paradigm shift. Indian J Orthod Dentofac Res.

[29] Baid R, Rathi N, Jain SA, Thosar N, Baliga S, Jayati M (2021). Comparison of effectiveness of diode laser with er: YAG laser on fluoride uptake of enamel surface using acidic and neutral topical fluorides: an in-vitro study. Int J Curr Res Rev.

[30] Singhania A, Borle A, Sathe S (2023). Efficacy of three different photobiomodulation therapies on primary and secondary implant stability in D3 and D4 bone type-a research protocol for randomised controlled trial. J Clin Diagn Res.

[31] Kumar AN, Jadhav V, Jawalekar R (2021). Light emitting diode mediated photobiomodulation therapy in orthodontics-a review of contemporary literature. J Evol Med Dent Sci.

[32] Kawasaki K, Shimizu N (2000). Effects of low-energy laser irradiation on bone remodeling during experimental tooth movement in rats. Lasers Surg Med.

[33] Limpanichkul W, Godfrey K, Srisuk N, Rattanayatikul C (2006). Effects of low-level laser therapy on the rate of orthodontic tooth movement. Orthod Craniofac Res.

[34] Youssef M, Ashkar S, Hamade E, Gutknecht N, Lampert F, Mir M (2008). The effect of low-level laser therapy during orthodontic movement: a preliminary study. Lasers Med Sci.

[35] Doshi-Mehta G, Bhad-Patil WA (2012). Efficacy of low-intensity laser therapy in reducing treatment time and orthodontic pain: a clinical investigation. Am J Orthod Dentofacial Orthop.

[36] Genc G, Kocadereli I, Tasar F, Kilinc K, El S, Sarkarati B (2013). Effect of low-level laser therapy (LLLT) on orthodontic tooth movement. Lasers Med Sci.

[37] Samantaray S, Sahu S, Gowd S, Srinivas B, Sahoo N, Mohanty P (2017). Speedy orthodontics: a review on methods of accelerating orthodontic treatment. Int J Oral Health Med Res.

[38] Sonpal PM, Mundada BP, Bhola ND, Kamble R, Mathew J (2022). Impacted mandibular first molar: a rare riddle. Cureus.

[39] Soma S, Iwamoto M, Higuchi Y, Kurisu K (1999). Effects of continuous infusion of PTH on experimental tooth movement in rats. J Bone Miner Res.

[40] Soma S, Matsumoto S, Higuchi Y, Takano-Yamamoto T, Yamashita K, Kurisu K, Iwamoto M (2000). Local and chronic application of PTH accelerates tooth movement in rats. J Dent Res.

[41] Collins MK, Sinclair PM (1988). The local use of vitamin D to increase the rate of orthodontic tooth movement. Am J Orthod Dentofacial Orthop.

[42] Al-Hasani NR, Al-Bustani AI, Ghareeb MM, Hussain S (2011). Clinical efficacy of locally injected calcitriol in orthodontic tooth movement. Int J Pharm Pharm Sci.

[43] Seifi M, Eslami B, Saffar AS (2003). The effect of prostaglandin E2 and calcium gluconate on orthodontic tooth movement and root resorption in rats. Eur J Orthod.

[44] Asiry MA (2018). Biological aspects of orthodontic tooth movement: a review of literature. Saudi J Biol Sci.

[45] Almpani K, Kantarci A (2016). Nonsurgical methods for the acceleration of the orthodontic tooth movement. Front Oral Biol.

[46] Kale S, Kocadereli I, Atilla P, Aşan E (2004). Comparison of the effects of 1,25 dihydroxycholecalciferol and prostaglandin E2 on orthodontic tooth movement. Am J Orthod Dentofacial Orthop.

[47] Diravidamani K, Sivalingam SK, Agarwal V (2012). Drugs influencing orthodontic tooth movement: an overall review. J Pharm Bioallied Sci.

[48] Ma D, Wang X, Ren X, Bu J, Zheng D, Zhang J (2020). Asperosaponin VI injection enhances orthodontic tooth movement in rats. Med Sci Monit.

[49] Jiao D, Wang J, Yu W (2022). Biocompatible reduced graphene oxide stimulated BMSCs induce acceleration of bone remodeling and orthodontic tooth movement through promotion on osteoclastogenesis and angiogenesis. Bioact Mater.

